# Clinical implications of lncRNA LINC-PINT in cancer

**DOI:** 10.3389/fmolb.2023.1097694

**Published:** 2023-03-17

**Authors:** Ihtisham Bukhari, Muhammad Riaz Khan, Fazhan Li, Bartlomiej Swiatczak, Rick Francis Thorne, Pengyuan Zheng, Yang Mi

**Affiliations:** ^1^ Henan Key Laboratory of Helicobacter pylori, Microbiota and Gastrointestinal Cancer, Marshall Medical Research Center, Fifth Affiliated Hospital of Zhengzhou University, Zhengzhou, China; ^2^ Research Center on Aging, Centre Intégré Universitaire de Santé et Services Sociaux de l'Estrie-Centre Hospitalier Universitaire de Sherbrooke, Sherbrooke, QC, Canada; ^3^ Department of History of Science and Scientific Archeology, University of Science and Technology of China, Hefei, China; ^4^ School of Environmental and Life Sciences, The University of Newcastle, Callaghan, NSW, Australia

**Keywords:** PINTology, LncRNA, LINC-PINT, Colon adenocarcinoma (CAC), Cancer biomarkers, Cancer therapy, Immune checkpoint inhibitors

## Abstract

Long noncoding RNAs (lncRNAs) possess the potential for therapeutic targeting to treat many disorders, including cancers. Several RNA-based therapeutics (ASOs and small interfering RNAs) have gained FDA approval over the past decade. And with their potent effects, lncRNA-based therapeutics are of emerging significance. One important lncRNA target is LINC-PINT, with its universalized functions and relationship with the famous tumor suppressor gene *TP53.* Establishing clinical relevance, much like p53, the tumor suppressor activity of LINC-PINT is implicated in cancer progression. Moreover, several molecular targets of LINC-PINT are directly or indirectly used in routine clinical practice. We further associate LINC-PINT with immune responses in colon adenocarcinoma, proposing the potential utility of LINC-PINT as a novel biomarker of immune checkpoint inhibitors. Collectively, current evidence suggests LINC-PINT can be considered for use as a diagnostic/prognostic marker for cancer and several other diseases.

## Introduction

Sequencing of the human genome has revealed that ∼20,000 traditional genes encode protein molecules, occupying just 2% of the total genetic landscape (Salzberg, 2018; Viggiano, 2022). The remaining genome consists of non-protein-coding regions, including numerous ‘RNA only’ genes, among which there is a large but diverse group called long non-coding RNAs or lncRNAs for short (Elkon and Agami, 2017; Dietlein et al., 2022; Paloviita and Vuoristo, 2022). Subsequent analyses have shown that lncRNAs fulfill various regulatory roles in healthy and cancerous tissues (Geng et al., 2018; Bi et al., 2019; Farzaneh et al., 2022a; Farzaneh et al., 2022b; Farzaneh et al., 2022c; Nasrolahi et al., 2022). As such, their expression and function are often altered in disease states (Kleinbrink et al., 2021; Yang et al., 2021; Anderson and Anderson, 2022; Dietlein et al., 2022; Erdogan et al., 2022) with selected examples now being used as diagnostic markers for various diseases including cancers (Li et al., 2019; Deng and Zou, 2022; Najafi et al., 2022; Zhang et al., 2022). Among these, one particular lncRNA called LINC-PINT (**P**53 **I**nduced **l**ong **N**on-coding **T**ranscript) appears exceptional because of its universalized functions and relationship to the famous tumor suppressor gene P53 (He et al., 2021; Bukhari et al., 2022). Indeed, like P53, LINC-PINT was first proposed as an oncogene but later revealed as a tumor suppressor in human cancers.

LINC-PINT contributes to a variety of biological processes impacting cancer cell growth and metastasis, with involvement in processes ranging from DNA damage responses to cell senescence and apoptosis (Simchovitz et al., 2020; He et al., 2021; Wang et al., 2021; Xiang et al., 2021; Zhang et al., 2021; Bukhari et al., 2022). It has also been shown to be an essential regulator of many other diseases, including autoimmune diseases (Wang and Zhao, 2020; Salviano-Silva et al., 2021). These functions arise through interactions affecting epigenetic regulation or direct interactions with other biomolecules, including proteins and other lncRNAs (Xu et al., 2019; Bukhari et al., 2022). Some alternatively spliced variants of LINC-PINT also act as host transcripts for circular RNA (circRNA) generation, and these can serve as sources for the translation of short functional peptides (Xiang et al., 2021). More than 100 alternatively spliced variants of LINC-PINT have been identified (Bukhari et al., 2022). Still, to date, only a few of these have been thoroughly studied—a feature earning the name PINTology to describe the current and future studies involving LINC-PINT.

## Therapeutic importance of lncRNA LINC-PINT

**FIGURE 1:**
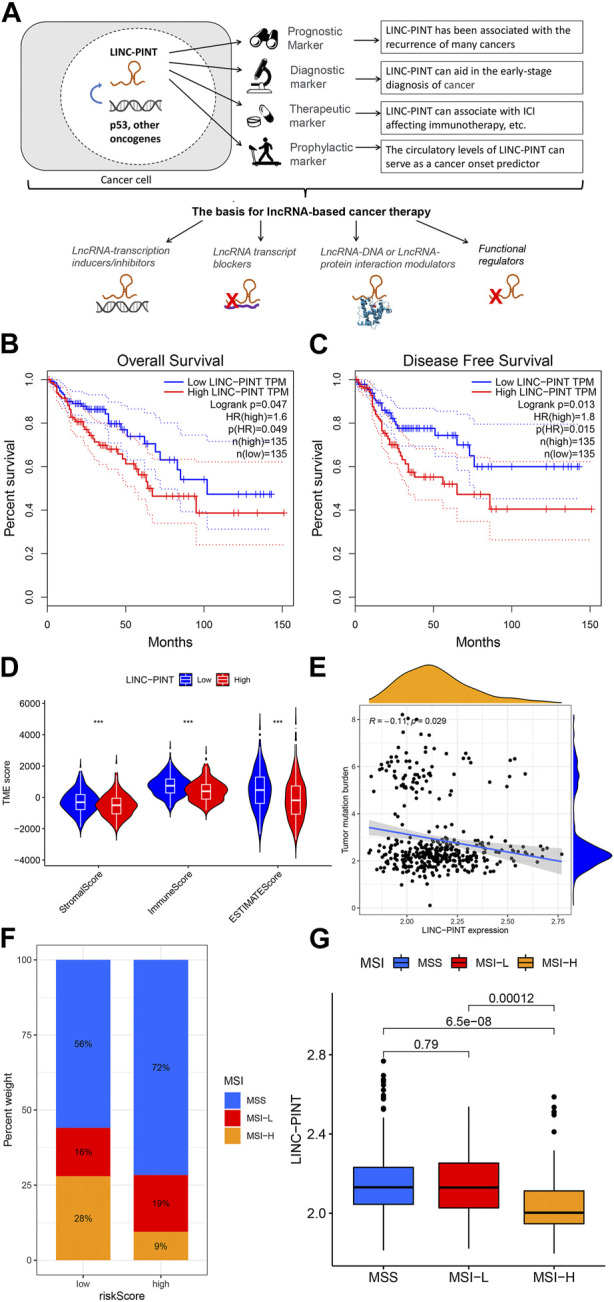
Clinical potentials of LINC-PINT. **(A)** Overview of the clinical applications of LINC-PINT. In short, it can be used as a prognostic marker to ascertain the course of cancer progression based on its expression level. It can also be used as a diagnostic marker to aid the early detection of ontological transformation. The third type of application, already in pre-clinical testing, predicts response to cancer therapy. Finally, less explored but equally important is the potential of LINC-PINT-based studies to determine the susceptibility of healthy subjects to cancer. These applications can be used, in turn, to tailor and administer suitable lncRNA-based cancer therapies. Correlation of LINC-PINT expression with **(B)** Overall survival and **(C)** Disease-free survival in Colon Adenocarcinoma **(D)** Association of LINC-PINT expression with immune scores, including stromal, immune, and ESTIMATE scores, **(E)** Tumor Mutation Burden, and **(F–G)** microsatellite instability in colon adenocarcinoma patients.

Cancer cells exhibit variability formally termed tumor heterogeneity, and this is a well-known explanation behind the failure of cancer therapy where resistant cells selectively survive repeated drug treatments (Brutovsky, 2022; Mahinfar et al., 2022). Contributing to this problem is also the lack of suitable diagnostic, therapeutic and prognostic biomarkers (Najafi, 2022). Hence, optimizing treatment outcomes for any disease is only possible with full knowledge of the underlying mechanisms. In this regard, recent studies involving lncRNAs have substantially contributed to understanding the cancer (Li et al., 2022b; Singh et al., 2022; Zhong et al., 2022). However, exploiting the potential of lncRNAs as biomarkers and therapeutic targets is just the beginning (Mirzaei et al., 2022). For example, lncRNAs associated with immune checkpoints (Li et al., 2022a; Jiang et al., 2022; Wen et al., 2022) have been used as disease biomarkers in the new wave of cancer immunotherapy that is currently revolutionizing oncology practice (Figure 1A). Nevertheless, small successes in the clinical setting are expected to help build confidence in lncRNAs as biomarkers and produce the momentum needed for their broader applications.

Although the tumor-suppressive function and downregulation of LINC-PINT in different cancers have been thoroughly established (He et al., 2021; Bukhari et al., 2022), its clinical use remains extremely limited. It is interesting to note that many of the vital protein targets of LINC-PINT involved in cell cycle regulation are already widely used in the diagnostic setting (Caputo et al., 2014; Xu et al., 2019). Furthermore, some of these are cancer treatments with inhibitors of LINC-PINT’s targets now being used in treating melanoma (George et al., 2019). As a starting point, the most appropriate clinical application of LINC-PINT would be as a diagnostic or predictive marker for specific cancers such as esophageal cancer, where LINC-PINT has been associated with cancer recurrence (Zhang et al., 2019; Rong et al., 2021). Indeed, the abnormal expression of LINC-PINT reflects many vital predictors of cancer outcomes such as lymph node metastasis, tumor size, and differentiation status (Figure 1A). Notably, the levels of LINC-PINT released by tumor cells into the blood could also be used to measure therapeutic responses, as specific anti-cancer drugs induce the re-expression of LINC-PINT. Other positive associations with neuropathy along with pemphigus foliaceous, diabetes, and arthritis may also be exploited, suggesting LINC-PINT as a multifaceted target for cancers and other diseases.

### Data collection

The complete gene expression profiles and clinical information of 473 colon cancer patients were downloaded from the TCGA online database (https://portal.gdc.cancer.gov/). The R software package “maftools” was used to calculate each sample’s tumor mutation burden (TMB). The microsatellite instability (MSI) score and groupings of the “TCGA-COAD” samples were downloaded using “TCGAbiolinks,” including: “MSI -H,” “MSI-L,” and “MSS.”

### Survival and immune function analyses

We analyzed the overall survival and disease-free survival of LINC-PINT in COAD within the online database GEPIA (http://gepia.cancer-pku.cn/). The Estimation of Stromal and Immune Cells in Malignant Tumors using the Expression Data (ESTIMATE) algorithm was used to determine the unique properties of the transcriptional profiles to infer the tumor cellularity as the tumor purity. The relationship of LINC-PINT expression, Stromal Score, and Immune Score with ESTIMATEScore was plotted as a violin plot using the “violin” package in the R program. Furthermore, we analyzed the correlations between different LINC-PINT expression groups, TMB and MSI with data presented as boxplots and scatterplots using “ggplot2”.

## Relationship between LINC-PINT and immune responses in colon adenocarcinoma (COAD)

We assessed the survival of patients and immune function associated with LINC-PINT expression in COAD tissues from the TCGA resource. Notably, patients whose tumors expressed low levels of LINC-PINT showed better overall and disease-free survival (Figure 1B, C). Subsequently, LINC-PINT showed a strong association with immune indicators, Tumor Mutation Burden (TMB), and Microsatellite Instability (MSI) in the COAD tumor microenvironment. COAD cases exhibiting higher expression of LINC-PINT had significantly lower immune scores, including stromal, immune, and ESTIMATE scores (Figure 1D), meaning that patients with low expression of LINC-PINT have a better immune response. Additionally, TMB and MSI have been used as prognostic markers for many cancers, especially for those receiving immunotherapy (Rizzo et al., 2021); thus, these can be used as predictive biomarkers for the efficacy of immunotherapy. Patients with low expression of LINC-PINT showed significantly higher TMB and MSI scores (Figures 1E–G), reflecting that LINC-PINT may be used as a novel biomarker of immune checkpoint inhibitors (ICIs).

### Clinical features of LINC-PINT in COAD

**FIGURE 2:**
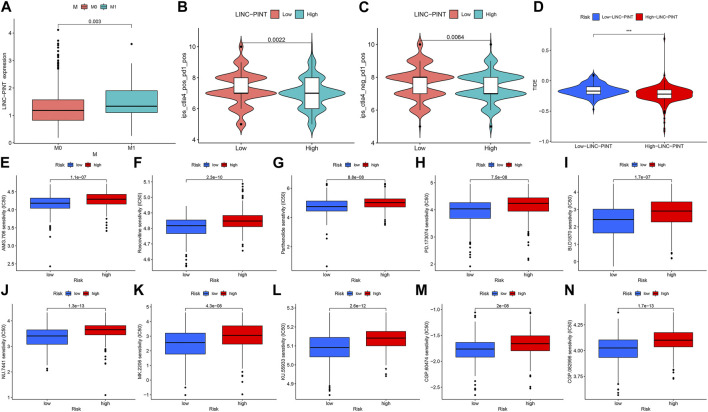
Relationship between LINC-PINT and clinical characteristics of patients with COAD. **(A)** Boxplot of differences in LINC-PINT expression levels between metastatic and non-metastatic groups of COAD patients. M0: non-metastasis; M1: tumor metastasis. **(B, C)** The immunophenotype scores in the low-LINC-PINT group and high-LINC-PINT group. **(D)** The TIDE score distribution between high- and low- LINC-PINT groups. **(E–N)** Drug sensitivity analysis of multiple chemotherapeutic drugs in the high LINC-PINT expression group and the low LINC-PINT expression group, respectively.

Additionally, we took advantage of the clinical characteristics and other clinical information of the 388 COAD cases available within the TCGA database. We stratified patients based on LINC-PINT expression in their primary tumors, dividing the samples into high-expression and low-expression groups. The relationship between LINC-PINT and the clinical features of COAD was then studied using the Wilcoxon rank sum test using *p* < 0.05 to denote statistical significance. Table 1 compares the differences between the high and low-expression LINC-PINT groups wherein high LINC-PINT expression showed a significantly higher proportion of tumor metastasis index (M1) cases compared to the low-expression group (*p* = 0.0357). Similarly, the boxplot analysis showed that the expression level of LINC-PINT was higher in patients with metastases (Figure 2A) (*p* = 0.003).

**TABLE 1:** Clinical features of the LINC-PINT in COAD. Out of the total of 473 COAD samples, we filtered out the complete clinical information of 388 cases from TCGA, and based on the LINC-PINT expression, the patients were divided into high and low-expression groups. Furthermore, patients were categorized based on their clinical characteristics (age, gender, T, N, M, stage, etc.) and compared the distribution of LINC-PINT high and low-expression samples in different groups. The proportion of M0 and M1 in the high-expression group was 80.2% and 19.8%, whereas in the low-expression group M0 and M1 proportion was observed as 88.48% and 11.52%, respectively. The distributions of metastasis-related clinical features were also significantly different than the control.

Covariates	Type	Total	High_LINC-PINT	Low_LINC-PINT	*p*-value
Age	≤65	152 (39.18%)	68 (34.52%)	84 (43.98%)	0.0711
Age	>65	236 (60.82%)	129 (65.48%)	107 (56.02%)	
Gender	FEMALE	184 (47.42%)	87 (44.16%)	97 (50.79%)	0.2284
Gender	MALE	204 (52.58%)	110 (55.84%)	94 (49.21%)	
Stge	Stge I-II	225 (57.99%)	111 (56.35%)	114 (59.69%)	0.573
Stge	Stge III-IV	163 (42.01%)	86 (43.65%)	77 (40.31%)	
T	T1-2	73 (18.81%)	31 (15.74%)	42 (21.99%)	0.1482
T	T3-4	315 (81.19%)	166 (84.26%)	149 (78.01%)	
M	M0	327 (84.28%)	158 (80.2%)	169 (88.48%)	0.0357
M	M1	61 (15.72%)	39 (19.8%)	22 (11.52%)	
N	N0	233 (60.05%)	116 (58.88%)	117 (61.26%)	0.8924
N	N1	88 (22.68%)	46 (23.35%)	42 (21.99%)	
N	N2	67 (17.27%)	35 (17.77%)	32 (16.75%)	

Furthermore, we validated the immune efficacy of LINC-PINT expression *via* the TCIA database. The IPS values were calculated based on immunogenicity from the TCIA database and then analyzed against LINC-PINT expression, finding higher effectiveness of immunotherapy in the low-risk group (*p* < 0.001) (Figures 2B, C). Moreover, Tumor Immune Dysfunction and Exclusion (TIDE) algorithm predicts ICB response and evaluates immune escape ability (http://tide.dfci.harvard.edu/). Therefore, we used the TIDE database to verify the relationship between risk scores and immune checkpoint inhibitors (ICIs) and found that the high LINC-PINT expression group has lower TIDE scores (Figure 2D). Together, this suggests that patients with high LINC-PINT expression have a lower possibility of immune escape than patients with low LINC-PINT expression, so they are more suitable for Immune Checkpoint Inhibitors (ICI) treatment.

### The relationship between LINC-PINT and anticancer therapies

In addition, we also analyzed the relationship between the expression of LINC-PINT and tumor sensitivity to immunotherapy and chemotherapy drugs. IPS-PD1/PD-L1 blocker and IPS-CTLA4 blocker data on COAD from the TCGA were obtained and analyzed using The Cancer Immunome Atlas database (TCIA, https://tcia.at/home) to predict the patients’ response to ICI as high- and low-risk groups. To explore the efficacy of chemotherapeutic drugs and their relationship with PINT, we used the pRRophetic package. Using the Wilcoxon test, we screened the chemotherapeutic drugs significantly different in the LINC-PINT expression group and displayed the IC^50^ distribution of different drugs in the two groups of samples through a boxplot (pFilter = 0.0001). We chose the top 10 chemotherapy drugs having highly significant differences in different risk groups and displayed the IC^50^ distribution of different drugs in the two groups of samples by boxplot (pFilter = 0.0001) (Figure 2E–N). The results showed that COAD patients with different expressions of LINC-PINT have apparent differences in sensitivity to various chemotherapeutic drugs, particularly patients whose tumors exhibit low LINC-PINT expression are more sensitive to chemotherapy.

## The future of lncRNA LINC-PINT

Compared to most lncRNAs, extensive literature exists for LINC-PINT, but a key question is whether this truly reflects most or, if not all, the underlying molecular mechanisms involved. In particular, the relationship between the functions of LINC-PINT in cancers *versus* non-malignant diseases needs to be better explored. For example, in viral hepatitis leading to liver cancer development, LINC-PINT foremost contributes to inflammation during viral infection and then later, to the pathogenic properties of the cancer cells (Khatun et al., 2021a; Khatun et al., 2021b), but whether the underlying processes involved are the same in both disease stages is not clear. Consequently, more intensive patient-based clinical studies are required to dissect these points. Furthermore, the large number of splice variants complicates this task, and it will be necessary to identify which variants share functions *versus* those that contribute to different functions. It is worth mentioning that at least one LINC-PINT variant produces small peptides, but this phenomenon’s frequency needs to be clarified. As functional products, these peptides may themselves serve as diagnostic molecules or treatment targets. It would be similarly valuable to ascertain more knowledge of the natural activators or inhibitors of LINC-PINT, which could be used to open potential new doors in cancer therapeutics. And to end on a sobering note, the number of lncRNA genes may rival or exceed the number of coding genes with LINC-PINT just one amongst thousands. However, there is the expectation that the pioneering studies involving LINC-PINT will provide important precedents for the clinical applications of lncRNAs.

## Discussion

LncRNAs are crucial regulators of various cellular pathways specifically cancer-associated pathways and use different mediators to function (Peng et al., 2017; Kopp and Mendell, 2018; Ali and Grote, 2020), while the majority of lncRNAs are functionally uncharacterized (Li et al., 2017; Lorenzi et al., 2021). Specifically, the regulation of lncRNAs occurs during specific occasions, such as development and cell growth (Sun et al., 2018). Mechanistically, lncRNAs bind with DNA, RNA, and proteins for transcriptional, post-transcriptional, and post-translational regulations (Schmitt et al., 2016; Hull et al., 2021; Yue et al., 2021). LncRNAs play vital roles in cancer initiation and progression or inhibition by affecting various pathways and expression of genes (Zhuo and Kang, 2017; Statello et al., 2021), showing that lncRNAs are either onco-suppressor or onco-promoter.

Similarly, LINC-PINT is a crucial lncRNA that mainly acts as an onco-suppressor in various cancers, but its clinical use is almost neglected. However, studies have suggested it as a potential biomarker for cancer prognosis (Ghafouri-Fard et al., 2021; He et al., 2021; Ghafouri-Fard et al., 2022). Through analysis of colon cancer data from the TCGA data, we determined an enticing relationship between LINC-PINT expression, prognosis and patients’ survival. Interestingly, patients with low expression of LINC-PINT showed better survival and significantly higher TMB and MSI scores together with better responses to chemotherapy, thus supporting the clinical importance of LINC-PINT, especially its use as a biomarker for monitoring responses to immunotherapy and chemotherapy.

## Conclusion

It is now commonly known that lncRNAs are key players in cancer regulation, a feature that promotes their application as target-based therapies in cancer and other relevant diseases. Interestingly, this promise has now been realized with several RNA-based therapeutics being recently approved by the FDA although their integration into the healthcare system still requires extensive clinical trials. It is well known that LINC-PINT has universalized tumor suppressor functions and a relationship to the tumor suppressor gene TP53 and several LINC-PINT targets are commonly used in clinical practice (directly or indirectly). Based on these facets, LINC-PINT emerges as a priority target for studying the clinical potential of lncRNAs (PINTology), especially for cancer therapeutics. However, the direct application of LINC-PINT is still limited due to the lack of clinical trials and clinicians’ confidence. Nevertheless, the current evidence proposes applications as a diagnostic marker for cancer and several other diseases with alterations in LINC-PINT circulatory levels providing a readily accessible platform to develop appropriate tests.
